# Long-term outcomes of bioprosthetic tricuspid valves: a systematic review of studies published over the last 20 years

**DOI:** 10.1093/ehjimp/qyaf097

**Published:** 2025-08-15

**Authors:** Edoardo Zancanaro, Michele Di Mauro, Giovanni Alfonso Chiariello, Daniel Sebastian Dohle, Karl-Patrik Kresoja, Jacopo Lin, Gaia De Angelis, Sebastian Rosch, Karl-Philipp Rommel, Michele De Bonis, Roberto Lorusso

**Affiliations:** Cardiovascular Research Institute, CARIM, University of Maastricht, Universiteitssingel 50, Maastricht, The Netherlands; University Hospital San Raffaele, Via Olgettina 60, Milano, Italy; Cardiovascular Research Institute, CARIM, University of Maastricht, Universiteitssingel 50, Maastricht, The Netherlands; Cardiovascular Research Institute, CARIM, University of Maastricht, Universiteitssingel 50, Maastricht, The Netherlands; Heart & Vascular Centre, Maastricht University Medical Centre, Universiteitssingel 50, Maastricht, The Netherlands; Department of Cardiovascular Sciences, Agostino Gemelli Foundation Polyclinic IRCCS, Rome 00136, Italy; Department of Cardiovascular Surgery, University Medical Center Mainz, Mainz 55131, Germany; Department of Cardiology, University Medical Center Mainz, Mainz 55131, Germany; University Hospital San Raffaele, Via Olgettina 60, Milano, Italy; Department of Cardiovascular Sciences, Agostino Gemelli Foundation Polyclinic IRCCS, Rome 00136, Italy; Department of Cardiology, University Medical Center Mainz, Mainz 55131, Germany; Department of Cardiology, University Medical Center Mainz, Mainz 55131, Germany; University Hospital San Raffaele, Via Olgettina 60, Milano, Italy; Cardiovascular Research Institute, CARIM, University of Maastricht, Universiteitssingel 50, Maastricht, The Netherlands; Heart & Vascular Centre, Maastricht University Medical Centre, Universiteitssingel 50, Maastricht, The Netherlands

**Keywords:** tricuspid valve, bioprosthetic valve, long-term outcomes, degeneration

## Abstract

Although the use of bioprostheses for tricuspid valve replacement (bTVR) has always been controversial in terms of results, their long-term durability is not well described. This systematic review aimed to identify, assess the quality, and review the outcomes in studies reporting on long-term outcomes after bTVR published over the last 20 years. Medline, Embase, and Cochrane CENTRAL were searched for studies reporting on at least five years of follow-up after bTVR. Cohort characteristics, definitions of structural valve deterioration (SVD), whose definition followed the criteria, and outcomes were summarized. The risk of bias in included studies was assessed using the Cochrane QUIPS tool. Ninteen studies, including 2541 patients were identified. The commonest implanted bioprosthesis was porcine-type with 1950 patients receiving this valve, followed by bovine pericardial (BP) type with 591 cases. Freedom from SVD in studies reporting outcomes up to 5–10 years ranged from 80% to 100% and in studies reporting to 15–20 years from 47% to 90%. Freedom from re-operation in studies reporting up to 5–10 years ranged from 94.7% to 100% and in studies reporting up to 15–20 years ranged from 49% to 95%. Reports of post-operative echocardiography were lacking, despite the heavy reliance on echocardiography for SVD diagnosis. There is considerable variability in reporting bTVR-related long-term outcomes. As such, it is difficult to generate an unbiased, generalizable understanding of long-term outcomes after bTVR across the spectrum of tricuspid disease phenotypes. Future clinical research will require more attention and detailed data report to fil such a gap.

## Graphical Abstract

**Graphical Abstract:**
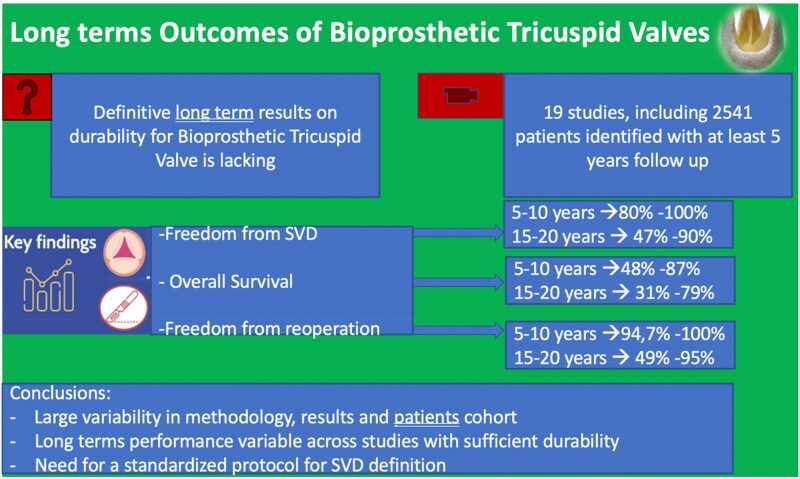

## Introduction

Bioprosthetic tricuspid valve replacement (TVR) has been introduced to mitigate the risk of thromboembolic and bleeding complications associated with mechanical valve prostheses and related treatment.^1^ However, bioprosthetic valves account for other type of valve-related shortcomings, particularly structural valve deterioration (SVD), that incorporates intrinsic valve failure, due to leaflet tears, calcification, fracture and stent creep, amongst others.^2,3^ SVD can lead to re-emergence of symptoms related to valvular heart disease, negatively affect prognosis, and may necessitate re-intervention once clinical condition as well cardiac imaging (echocardiography in the first instance) indicate haemodynamically significant prosthetic dysfunction.^3,4^ Despite several changes and advanced valve design and biological tissue-related features, risk of SVD at variable time from surgery remain a post-operative shortcoming requiring adequate patient monitoring.^3^ As such, defining the natural history and exploring the risk factors of SVD is paramount in informing prosthesis choice in patients undergoing TVR. This might also represent a critical source of information to understand and improve bioprosthetic tricuspid valve durability. However, the long-term durability of tricuspid bioprostheses in current clinical use has been poorly investigated and clear information are missing. Thus, a systematic review of studies reporting the outcomes of TVR with bioprostheses published over the past 20 years was conducted to investigate the heterogeneity in outcome definitions, SVD ascertainment and follow-up methodology and duration in patients submitted to such a cardiac valve replacement for variable underlying aetiologies of tricuspid valve dysfunction.

## Methods

This systematic review was carried out according to the Preferred Reporting Items for Systematic Reviews and Meta-analysis statement.^5^

### Eligibility criteria

All studies fulfilling the following criteria were included: reporting on TVR in adult humans, written in English, throughout the last 20 years (from 1992 to 2023), presenting data with follow-up of at least 5 years, with no restriction of maximal follow-up time. Studies reporting at least one of the following outcomes were selected: long-term survival, freedom from TV re-operation or TV-related SVD diagnosis. Studies with the following criteria were excluded: studies on children or non-human subjects, transcatheter tricuspid valve implantation and case reports/commentaries/letters to the editor/conference abstracts. In cases of multiple publications using the same database, the one with the longest patient-year follow-up and completeness of data was included.

### Information sources

Searches were conducted in Embase, Medline, and Cochrane CENTRAL. Furthermore, references from included publications and from the main cardiac surgery specialty journals (*European Journal of Cardiothoracic Surgery*, *Annals of Thoracic Surgery*, and *Journal of Thoracic and Cardiovascular Surgery*) over the past 20 years were also screened. The search was performed until 18/09/2024.

### Search strategy

After the initial search, secondary search was supplemented for the named valve types identified. The search strategy was discussed and agreed among all authors.

### Selection process

Independent reviewers (E.Z. and M.D.M.) screened abstracts and subsequently full-texts of studies. Disagreement was resolved by discussion with the senior author (R.L.).

### Data collection process

Data were extracted to Microsoft Excel by independent reviewers that subsequently checked each other's entries and data integrity.

### Data abstraction/synthesis

Data items collected included: study period, number of patients, name of prosthesis used, size of prosthesis, SVD definition used, follow-up time, completeness of follow-up, mean age, sex, aetiology of tricuspid pathology, prosthetic effective orifice area (EOA), mean gradient across the prosthesis, concomitant procedures, presence of atrial fibrillation, body surface area, diabetes mellitus, insulin use, pre-op NYHA, peripheral vascular disease, chronic obstructive pulmonary disease (COPD), neurological comorbidity pre-operatively, chronic kidney disease pre-operatively, EuroSCORE (logistic) or STS score if available, pre-operative left ventricular ejection fraction (LVEF), cardiopulmonary bypass (CPB) time, and cross clamp (XC) time.

Primary outcomes extracted included: late survival (actuarial survival at the longest follow-up point), re-intervention (actuarial freedom from re-intervention at the longest follow-up point), and SVD (actuarial freedom from SVD at the longest follow-up point). Other outcomes collected included: annualized event rates for thromboembolic events, endocarditis, and major haemorrhagic events.

### Risk of bias assessment

The Cochrane QUIPS tool was used to assess the risk of bias (four) against the following domains: study participation, study attrition, prognostic factor measurement, outcome measurement, study confounding, statistical analysis and reporting. Domains were scored as low, moderate, or high risk of bias. Where two or more high scores were given, the overall risk of bias was graded as high. If five or six domains were low, a low overall score was given. Anything in between was given a moderate score.

No ethical approval was necessary due to the retrospective analysis.

## Results

**Figure 1:**
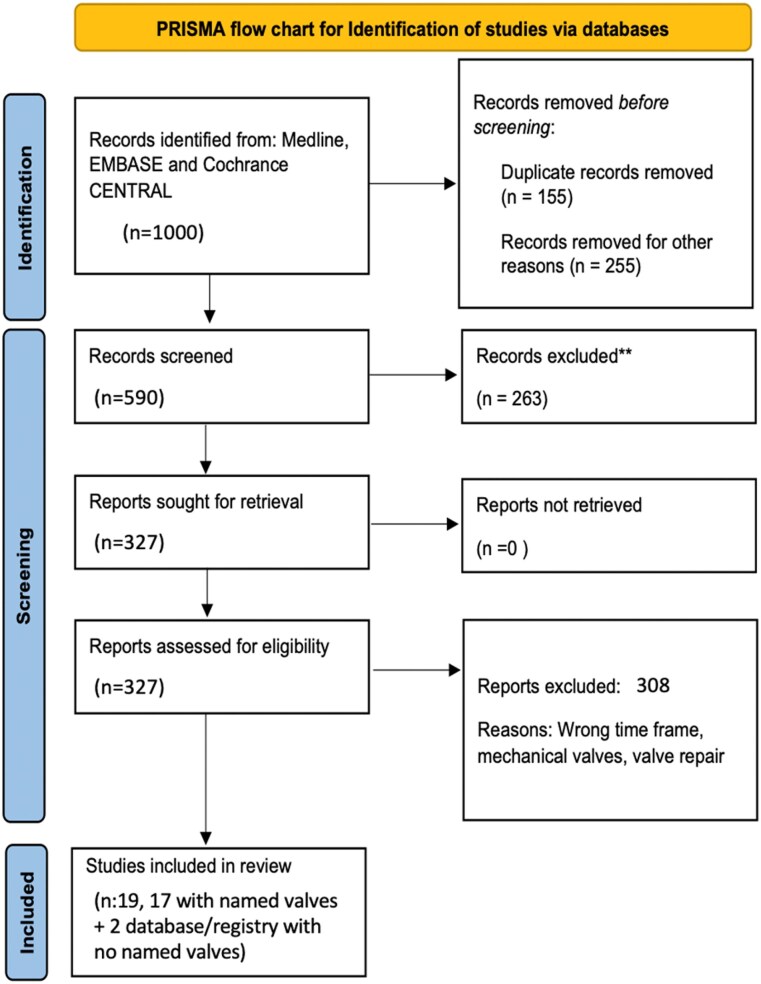
PRISMA flow chart for identification of studies via databases.

Nineteen studies were identified (*Figure 1*), including 17 studies reporting on specific valve models and two studies that used International Classification of Disease (ICD) codes to identify valve surgery patients from administrative/institutional databases. These did not ascertain valve models or SVD. The reports focussed on bioprostheses that accounted for four different types: Carpentier Edwards Pericardial (Edwards Life-Science, CA, USA), Epic (Abbott, Minneapolis, USA), Hancock I (Medtronic, Minneapolis, USA), Hancock II (Medtronic, Minneapolis, USA), and the Mosaic valve (Medtronic, Minneapolis, USA), with the last three being constituted by porcine valves, and the first by bovine pericardium. These 19 studies included 2541 patients across seven countries with tissue valves implanted from 1975 to 2020.

**Figure 2:**
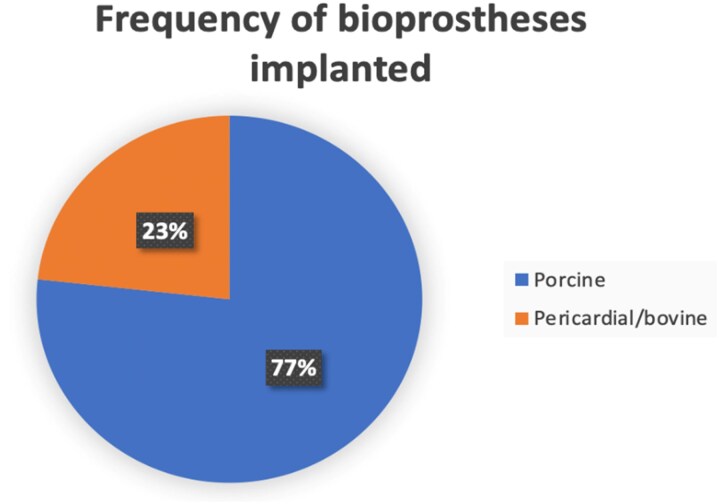
Frequency of bioprostheses implanted (%) in included studies.

Among the implanted tissue valves, the commonest bioprosthesis was porcine-type, with 1950 patients receiving this valve, followed by bovine pericardium (BP) with 591 (*Figure 2*). Within the porcine-type patient group, 448/1950 (23%) were Hancock II. In BP group, instead, the vast majority (90%) was Carpentier-Edwards bioprosthesis. The 65% of studies reporting bioprosthesis size, the commonest one was 31 mm, respectively.

**Figure 3:**
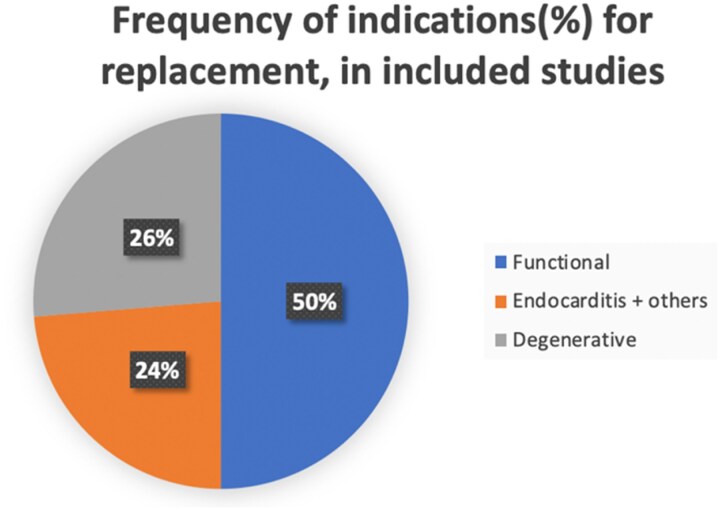
Frequency of indications (%) for replacement, in included studies.

Three studies (16%) did not report the underlying pathologies that necessitated valve replacement. Where the underlying pathology was reported in five studies, the method of how the pathology was ascertained was unclear in most studies. Where the pathology was reported (12 studies), 57% of implantations were for functional disease, 30% for degenerative, 27% for endocarditis, and the rest for other indications such as traumatic and mixed pathology (*Figure 3*). Almost a third of procedures (27.7%) in these studies were a re-operation.

Up to two-thirds of included patients had at least one concomitant procedure. Where reported (15), 28% had multiple concomitant procedures and isolated concomitant CABG was instead performed in 8% of the cases.

In terms of pre-operative profile, some variables were better documented, such as sex (female in 55%), persistent/permanent AF (in 30% of cases), and NYHA class status (III–IV in 47.3% of patients). Information was very limited on body surface area and concurrent medical therapy (for example the use of diuretics, anticoagulants/antiplatelets, AF therapy), as well as for right side heart failure symptoms. Very few findings have been reported for pre-operative echocardiography but LVEF has been reported in 10/19 studies, with a mean 53% ± 45–55.

In terms of post-operative echocardiography, post-operative mean gradient was only available in one study (4.7 mmHg mean, 3.5–5 mmHg range). Reported mean/median follow-up times ranged from 5 to 16 years and Kaplan–Meier reports of follow-up up of up to 20 years where available.

### Main outcomes

**Figure 4:**
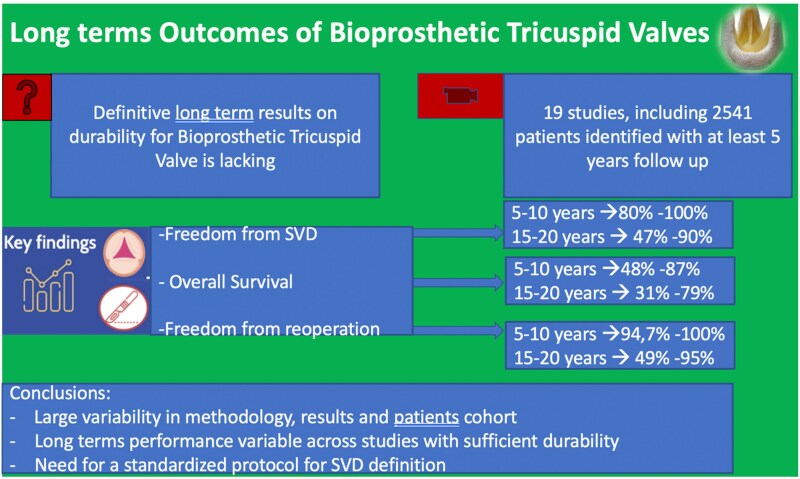
Central illustration.

Actuarial long-term survival in studies reporting a maximum of 5–10-year follow-up ranged from 48% to 87.5%. In studies reporting a 15–20-year follow-up, survival ranged from 31% to 79% (*Table 1*, *Figure 4*).

**Table 1:** Main outcomes for included studies with named valves

Author	Pub year	Study dates	Valve	Patients (*n*)	Mean (SD) age	Mean follow-up (y)	SVD, *n*	Longest follow-up (y)	Actuarial survival (%)	Actuarial freedom from SVD (%)	Actuarial freedom from re-operation (%)
Buzzatti N	2014	1997–2012	Porcine/bovine	50	63	7.4	4	10	79.4	74	87
Sohn SH	2023	2002–2018	Porcine/bovine	185	68	10		10	57.1		99
Kuwaki K	1998	1976–1994	Porcine	26	38	16	5	16	78	47	47
Filsoufi F	2004	1985–1999	Porcine	34	61	5	1	5	45	80	
Yuan Y	2023	2009–2020	Porcine	46	63	5		10	54	81.5	55.3
Jegaden O	1992	1975–1990	Porcine/bovine	42	53	15	11	15	83	4	95
Songur CM	2014	1993–2011	Porcine/bovine	62	61	12	1	12	61	99	99
Kawachi Y	1992	1975–1981	Porcine	23	36	16	2	15		94	78
Patlolla SH	2022	1993–2018	Porcine	849	68	9		10	34	75	95
Chang BC	2005	1978–2003	Porcine	35	44	16		16	73	60	50
Anselmi A	2015	1971–2012	Porcine	155	57.4	15	17	15	36	85	84
Garatti A	2012	1980–2005	Bovine								
Kaplan M	2002	1980–2000	Porcine	32	33	12		15	85	90	90
Glower	1995	1974–1993	Porcine	129	53	10		15	31	49	49
Nakano K	1996	1985–1994	Bovine	67	52	10		10	75	71	71
Sala A	2022	1997–2020	Not specified	129	68	5		5	85	99	95
Tokunaga S, R.	2008	1995–2004	Porcine/bovine	14	38	15		5		91	92
Di Mauro	2019	1979–2018	Not specified	72	47	20		10	66		
Liu P	2021	1999–2018	Porcine/bovine	67	42	14		5	84	91	91

SVD, structural valve deterioration.

SVD based on explants only.

Only cumulative frequencies given.

Kaplan–Meier curves available but does not give exact figures in text or tables.

In studies reporting outcomes up to 5–10 years, freedom from SVD ranged from 80% to 100%, and in studies reporting 15–20-year follow-up, it ranged from 47% to 90%. Freedom from re-operation in studies reporting up to 5–10 years ranged from 94.7% to 100% and in studies reporting up to 15–20 years ranged from 49% to 95% (*Table 1*).

Thromboembolic events was hardly reported in details, but, when available, it ranged from 0.1% to 5%/year and endocarditis from 0% to 5%/year (*Table 1*).

### SVD definitions

Eight out of the included studies have made specific mention of SVD definition, mainly as proposed by Akins *et al*.^3^ Eleven studies relied purely on transthoracic echocardiography demonstrating severe haemodynamic deterioration [mean gradient across the prosthesis of >5 mmHg or an effective regurgitant orifice area (EROA) of >40 mm^2^].

In studies using the consensus criteria (Akins or Edmunds), there is no clear and specific mention of how SVD diagnoses where made based on the different components of the definition (how many on autopsy, examination of explant or clinical investigation/echocardiography).

### Porcine valves

Fifteen over 17 studies reporting SVD of porcine bioprosthesis were identified, and specifically five described Hancock II bioprosthesis (1329 patients), with the remaining 10 reporting the general classification only. Of these studies, three studies only reported outcomes up to 15 years of follow-up.

The longest follow-up periods reported in studies of patients receiving porcine valves ranged from 8 to 16 years. In studies reporting porcine valve outcomes to 10 years, freedom from SVD ranged from 47% to 99% and freedom from re-operation ranged from 49% to 99%.

The largest study on the Hancock I–II valve followed-up 129 patients revealing a 10-year survival of 48% ± 5% giving an actuarial freedom from re-operation for SVD at 10 years of 93% ± 4%. The SVD freedom instead was 92% ± 4%. At 15 years, the overall survival was 31% ± 9% and freedom from re-operation was 49% ± 17% and freedom from SVD was 49%.

### Bovine pericardial valves

Eight studies reported on the outcomes of the Carpentier-Edwards pericardial tricuspid bioprosthesis (591 patients). One study reported outcomes at 15 years and three up to 10 years. At 10–15 years, the reported range of freedom from SVD was 71–99%.

The study by Jegarden *et al*. provided the largest cohort of the Carpentier-Edwards pericardial valve in 331 patients demonstrating an actuarial survival of 56% and re-operation due to SVD of 90%.

### Database/registry data

Two studies were identified where only the type of valve (tissue) was clear, but not the model of the valve; 201 patients were seen and the mean follow-up ranged from 5 to 20 years. Ten-year survival was 66%, and freedom from re-operation for SVD at 5 years was 95% ± 4%. One study reported 20-year long-term survival that was 60%. No SVD was reported.

### Use of echocardiography to ascertain SVD

Despite the reliance of echocardiography to a large extent in identifying and describing SVD, no study made explicit use of a core laboratory for TTE or TEE evaluation, and there was a large variability in the number of patients who underwent one or more post-operative echocardiograms (range 35–100%). In most part, TTE follow-up (or other imaging) seems to have not been performed systematically over the whole follow-up period in these studies.

## Discussion

This review presents an up-to-date review of all studies reporting on the long-term outcomes of bioprosthetic valves implanted at tricuspid position over the past 20 years. We identified 19 studies, reporting on the outcomes of more than two-thousand patients with a mean follow-up of 10 years.

The key observations from our review are that (i) long-term results/SVD and related information of implanted tricuspid valve bioprostheses have been inconsistently reported, as shown by the limited available follow-up duration and published data, (ii) there is a large heterogeneity in the definition and ascertainment SVD, and (iii) a large variability in the characteristics of the cohorts studied. These issues make it difficult to generate a clear and comprehensive understanding of the performance and durability of tricuspid bioprosthesis surgically implanted.

### SVD finding and definition

Even though most of the studies referenced the definition from Akins *et al*.,^3^ it is very difficult to define properly when and how the different components (autopsy, explant and investigation) where used to define SVD. Furthermore, the majority of studies relied on major haemodynamic features to diagnose SVD (EROA > 40 mm^2^ and mean gradients of >8 mmHg). Some studies have even focussed on a different perspective, like freedom from re-operation.^1^ Nevertheless, using re-operation or freedom from re-operation as a sole marker of SVD, the risks of excluding patients with actual presence of SVD, but not reoperated, might be high and some overlap or missing can be found if considered advanced bioprosthetic valve failure.

The multi-society consensus of SVD in the aortic position makes a clear distinction between morphological changes of valve deterioration, moderate and severe haemodynamic effects.^6^ This description/differentiation is lacking as far as tricuspid bioprostheses is concerned. This obviously calls for a more structured approach to SVD in the tricuspid prosthesis to be developed and used in published series describing post-discharge bioprosthetic performance and durability, in similar manner to the TAVR/SAVR field.

A very important flaw in the analysed studies was the lack of haemodynamic/echocardiographic findings as well as the little evidence of systematic echocardiographic surveillance. Also, there was no explicit use of a core laboratory and the post-op TTE schedules were highly variable.

### Variability in the cohorts

Age at implantation remains the strongest known independent predictor of bioprosthetic valve longevity.^4^ In this review, mean age at implantation ranged from 33 to 68 years, revealing a degree of youth in the population treated not usually seen in other cardiac valve settings. As proof of this, the mean age of was 49.7 years. In cohorts with older patients, the competing risk of mortality might mask the emergence of SVD and without a sufficiently long follow-up and large cohort. Also, estimation of freedom from valve deterioration might become confounded as not enough patients survive long enough to experience the outcome.

Interestingly enough, the difficulties to find a precise spectrum of valve types, may question whether durability of one valve is purely based on the age group where it was studied or if intrinsic valve durability based on the valve type are at play as well.

Another major source of heterogeneity in the tricuspid valve durability literature is related to the endocarditis cohort.^7^ This is a very critical aspect since 122 patients from 10 studies reported endocarditis as aetiological agent of tricuspid pathology. Not much has been denoted at follow-up with only one study,^8^ reporting the 20% of follow-up endocarditis for pericardial bioprostheses.

### Limitations

To our knowledge, this is the most up-to-date review on the topic and focusses on the last 20 years to maximize the relevance of findings.

The current review presents some limitations.

First, we only included studies published in the English language since 1992. There is a chance that this will have introduced an element of publication bias.

Secondly, very few studies reported with clear precision the long-term echocardiographic results.

Thirdly, lots of paper selected were very old, in order to obtain long-term follow-up, but this brings to have some degree of lack of the new parametric analysis of right ventricle and tricuspid valve.

Lastly, precise clinical follow-up at more than 10 years was frequently missing and some degree of bias can be seen.

## Conclusion

There is now more than ever, particularly with the recent introduction of transcatheter biological valve implantation at tricuspid position, a need to enhance our understanding of bioprosthetic tricuspid valve durability and long-term outcomes. Ratifying the real-world outcomes of surgical bioprosthetic tricuspid replacement should form the gold standard against which transcatheter tricuspid valve interventions will be judged. As such, an unbiased, comprehensive understanding of long-term outcomes and predictors of valve degeneration across the spectrum of tricuspid disease phenotypes is needed.

## Supplementary Material

qyaf097_Supplementary_Data

## Data Availability

All data used to compile this review will be available upon reasonable request to the authors. The search strategies are available as Supplementary material.

## Lead author biography

Edoardo Zancanaro is a cardiac surgery resident who graduated in International Medical Program at Università Vita Salute San Raffaele in Milan, Italy, and he is currently resident at San Raffaele Hospital in Milan. He is also performing an advanced fellowship in minimally invasive cardiac surgery and Interventional Cardiology at University Hospital in Mainz. The principal goal is to become a hybrid cardiac surgeon with a specific attention to valvular heart diseases. The major topics of interest are minimally invasive/robotic CS and transcatheter valvular therapy with a close attention to new device/AI-incorporated tools to address valvular pathology. Edoardo is particularly interested in scientific education, and the podcast and broadcast that is leading for EHJIMP and ESC are the main vectors of it.
