# Supplementary material for: Long-term outcomes of bioprosthetic tricuspid valves: a systematic review of studies published over the last 20 years
Source: Eur Heart J Imaging Methods Pract. 2025 Aug 15;3(4):qyaf097. doi: 10.1093/ehjimp/qyaf097 (PMC12596361; doi:10.1093/ehjimp/qyaf097)
Supplement: qyaf097_Supplementary_Data [file qyaf097_supplementary_data.docx]

**Supplemental Methods**

Search strategy

Medline

*((((("heart valve prosthesis"/ and "tricuspid valve"/) or (tricuspid adj6 valve* adj6 (replace* or transplant* or xenotransplant* or xenograft* or heterotransplant* or heterograft* or prosthe* or bioprosthe* or stent*)).ab,ti.) and (heterografts/ or (xenograft* or xenotransplant* or heterograft* or heterotransplant* or ((xeno* or hetero* or porcine* or swine or pig or bovine* or nonhuman or animal or calf or cow) adj6 (graft* or transplant* or prosthe* or bioprosthe* or valve* or tricuspid))).ab,ti.)) not (exp animals/ not humans/)) and ("Clinical Trial".pt. or exp "Case-Control Studies"/ or "Intervention Studies"/ or exp "Longitudinal Studies"/ or exp mortality/ or mortality.xs. or exp "treatment outcome"/ or survival/ or "graft survival"/ or "quality of life"/ or (clinical* or trial* or prospect* or retrospect* or longitudin* or mortali* or outcome* or failure* or surviv* or (quality adj3 life) or result* or (follow* adj up*) or "long term" or longterm or death or evaluat* or effectiv* or reoperat*).ab,ti.) and english.la.) not (congresses or Letters or Notes or Editorials).pt.*

Embase Results

*(("heart valve prosthesis"/ AND "tricuspid valve"/) OR (tricuspid ADJ6 (valve*) ADJ6 (replace* OR transplant* OR xenotransplant* OR xenograft* OR heterotransplant* OR heterograft* OR prosthe* OR bioprosthe* OR stent*)).ab,ti.) AND (heterografts/ OR (xenograft* OR xenotransplant* OR 1heterograft* OR heterotransplant* OR ((xeno* OR hetero* OR porcine* OR swine OR pig OR bovine* OR nonhuman OR animal OR calf OR cow) ADJ6 (graft* OR transplant* OR prosthe* OR bioprosthe* OR valve* OR tricuspid))).ab,ti.) NOT (exp animals/ NOT humans/) AND ("Clinical Trial".pt. OR exp "Case-Control Studies"/ OR "Intervention Studies"/ OR exp "Longitudinal Studies"/ OR exp mortality/ OR mortality.xs. OR exp "treatment outcome"/ OR survival/ OR "graft survival"/ OR "quality of life"/ OR (clinical* OR trial* OR prospect* OR retrospect* OR longitudin* OR mortali* OR outcome* OR failure* OR surviv* OR (quality ADJ3 life) OR result* OR (follow* ADJ up*) OR "long term" OR longterm OR death OR evaluat* OR effectiv* OR reoperat*).ab,ti.) AND english.la. NOT (congresses OR Letters OR Notes OR Editorials).pt.*

Cochrane Central Results

*((tricuspid) AND (valve)) NEAR/6 (replace* OR transplant* OR xenotransplant* OR xenograft* OR heterotransplant* OR heterograft* OR prosthe* OR bioprosthe* OR stent*):ab,ti AND ((xenograft* OR xenotransplant* OR heterograft* OR heterotransplant* OR ((xeno* OR hetero* OR porcine* OR swine OR pig OR bovine* OR nonhuman OR animal OR calf OR cow) NEAR/6 (graft* OR transplant* OR prosthe* OR bioprosthe* OR valve* OR tricuspid*):ab,ti) NEAR/3 (valve* OR bioprosthe* OR prosthe*)):ab,ti) AND ((clinical* OR trial* OR prospect* OR retrospect* OR longitudin* OR mortali* OR outcome* OR failure* OR surviv* OR (quality NEAR/3 life) OR result* OR (follow* NEXT/1 up*) OR 'long term' OR longterm OR death OR evaluat* OR effectiv* OR reoperat*):ab,ti*
